# The NT5DC family: expression profile and prognostic value in pancreatic adenocarcinoma

**DOI:** 10.7150/jca.85811

**Published:** 2023-07-16

**Authors:** Xiaoqian Yu, Ru Sun, Xuejie Yang, Xiaoyun He, Hongbin Guo, Chunlin Ou

**Affiliations:** 1 Department of Pathology, Xiangya Hospital, Central South University, Changsha 410008, Hunan, China.; 2 Department of blood transfusion, Affiliated Hospital of North Sichuan Medical College, Nanchong 637000, Sichuan, China.; 3 Departments of Ultrasound Imaging, Xiangya Hospital, Central South University, Changsha 410008, Hunan, China.; 4 Department of Orthopedics, Xiangya Hospital, Central South University, Changsha 410008, Hunan, China.; 5 National Clinical Research Center for Geriatric Disorders, Xiangya Hospital, Central South University, Changsha, China.

**Keywords:** NT5DC gene family, pancreatic adenocarcinoma, immune infiltration, prognosis, biomarkers

## Abstract

Pancreatic adenocarcinoma (PAAD) is a malignant tumor with high morbidity and mortality rates. The NT5DC family is an evolutionarily-conserved family of 5'-nucleosidases that catalyze the intracellular hydrolysis of nucleotides. Although the NT5DC family has been linked to the initiation and growth of several cancers, its function in PAAD remains unclear. A series of bioinformatic analyses was used to ascertain the expression, prognosis, gene changes, functional enrichment, and immune regulatory functions of the NT5DC family in PAAD. NT5C2 and NT5DC1/2 mRNA and protein levels are increased in PAAD. Furthermore, the high mRNA expressions of NT5C2, NT5DC2, and NT5DC4 indicate a poor prognosis in patients with PAAD. The enrichment of biological processes and gene expression in the NT5DC family in PAAD were investigated using Kyoto Encyclopedia of Genes and Genomes and Gene Ontology analyses. Further investigations into immune infiltration revealed a close relationship between NT5DC gene expression and immune cell infiltration. These findings provide new insights into the biological function and prognostic value of the NT5DC gene family in PAAD.

## Introduction

Pancreatic adenocarcinoma (PAAD) is one of the most prevalent cancers worldwide 1, 2. Due to its poor prognosis, PAAD accounts for almost as many deaths as cases and is the fourth most common cause of cancer death in both sexes 3. Both the incidence and mortality of pancreatic cancer have increased over the past decade compared with those of most other solid tumors 4. The management of PAAD has evolved with the introduction of new surgical and therapeutic techniques, such as laparoscopic techniques and neoadjuvant chemoradiotherapy. However, only modest improvements in outcomes have occurred 5. To move toward an era of precision medicine, the identification of new biomarkers and therapeutic targets for PAAD are necessary 6, 7.

The NT5DC gene family consists of NT5C2, NT5DC1, NT5DC2, NT5DC3, and NT5DC4. The NT5DC family is an evolutionarily-conserved family of 5′-nucleotidases that catalyze the hydrolysis of nucleotides in the cell 8. Dysfunctions of members of the NT5DC family have been associated with disorders of the immune system, sensitivity to cancer therapy, and metabolic disorders 9. NT5C2 is responsible for the final dephosphorylation of 6-hydroxypurine monophosphates, including IMP, dIMP, GMP, dGMP, and XMP, before their extracellular export and plays a key role in the maintenance of the balance of nucleotides, nucleosides, and free groups in the purine library of the brain and spinal cord 10. Single-nucleotide polymorphisms in NT5DC1 have been associated with susceptibility to chronic obstructive pulmonary disease 11. Guo et al. 12 reported that NT5DC2 promotes the tumorigenicity of glioma stem cell-like cells via the upregulation of Fyn. Li et al. reported that NT5DC3 can be used as a biomarker to discriminate patients with type 2 diabetes (T2D) and T2D-induced colon cancer from healthy volunteers by detecting the expression level of NT5DC3 in the blood 13. In addition, NT5DC3 may be used as target of a novel cancer preventive treatment strategy, especially in patients with T2D who are prone to colorectal cancer (CRC) 14. Jin et al. 15 reported that suppressing NT5DC2 inhibits the metastatic progression of non-small cell lung carcinoma (NSCLC) by regulating the p53 signaling pathway. The reduced RNA binding protein IGF2BP2 down-regulates NT5DC2, which suppresses cell proliferation and induces cell cycle arrest and apoptosis in diffuse large B-cell lymphoma cells via the regulation of the p53 pathway 16. NT5C2 methylation regulates the interaction between DNMT1 and the insulin receptor in patients with T2D 17. Kulkarni et al. found that NT5C2 and AMPK activity in patients with T2D and obesity may be important for controlling insulin action and lipid metabolism in skeletal muscles 18. Previous studies have shown that NT5DC family members are associated with tumor progression 19, 20 and can serve as tumor markers 21, 22. However, the expression, prognosis, and immune properties of the NT5DC family in PAAD have not been comprehensively analyzed. In this study, the expression of NT5DC family genes and their association with clinical characteristics of PAAD were analyzed using research databases and bioinformatics tools. The results of this study provide new insights into the prognostic value and biological role of the NT5DC family in PAAD.

## Materials and methods

### Gene Expression Profiling Interactive Analysis 2

Gene Expression Profiling Interactive Analysis 2 (GEPIA2) has been used to study various genes in different cancer types and identify potential biomarkers and therapeutic targets using differential expression analysis, survival analysis, and similar gene identification 23. The GEPIA2 database was used to analyze the expression of members of the NT5DC family of genes in 171 samples of normal pancreas and 181 samples of PAAD.

### University of Alabama at Birmingham Cancer Data Analysis Portal

The University of Alabama at Birmingham Cancer Data Analysis Portal (UALCAN) is an *in silico* validation platform for target genes that can help identify relevant genes for biomarker analysis, expression profile analysis, and survival analysis 24. In this study, the "individual cancer stage" model was used to analyze the relationships between the mRNA and protein expressions of different NT5DC family members and clinicopathological parameters in PAAD. A student's t-test was used to compare the results, and *p* < 0.05 was considered to be statistically significant.

### cBioPortal

The cBioPortal (http://cbioportal.org) is an online portal for the exploration, visualization, and analysis of cancer genomic data 25. A map from The Cancer Genome Atlas (TCGA) and 186 cBioPortal pancreatic cancer datasets of NT5DC gene families, which included mutations and mRNA expression information, were used in this study. The threshold for the absolute value of the log2 fold-change was 1.5. The *p*-value cutoff was set at 0.05.

### Cytoscape

Cytoscape (http://cbioportal.org) allows for the integration of molecular interaction networks with expression data and other molecular states into one framework 26. Cytoscape was used to selectively integrate 208 genes that were functionally co-expressed with members of the NT5DC family. The degrees to which proteins interact with each other are represented by the sizes of the nodes.

### Metascape

Metascape combines functional enrichment, interactome analysis, gene annotation and membership search. It uses more than 40 independent knowledge bases within one integrated portal 27. Metascape was used for Kyoto Encyclopedia of Genes and Genomes (KEGG) and Gene Ontology (GO) pathway enrichment analyses of the NT5DC family.

### Tumor Immune Estimation Resource

The Tumor Immune Estimation Resource (TIMER2.0, http://timer.cistrome.org/) is a more robust estimation of immune infiltration levels for TCGA or user-provided tumor profiles that uses six state-of-the-art algorithms 28. TIMER2.0 provides four modules with which to explore associations between immune infiltrates and genetic or clinical features and cancer-related associations in the TCGA cohorts 29. The TIMER2.0 database was used to investigate the relationship between NT5DC family members and immune cell infiltration and the associations between the NT5DC family and B cells, CD8+ T cells, neutrophils, macrophages, dendritic NK-cells, Th1-cells, Treg-cells, and monocytic markers.

### Kaplan-Meier Plotter Database

To integrate gene expression information and clinical prognostic data for the meta-analysis and to discover and validate survival-related molecular markers, the Kaplan-Meier Plotter database (https://kmplot.com) was used. Using an automatic selection of the best cutoff value, the 177 PAAD samples were divided into high- and low-expression groups, and the expressions of members of the NT5DC family were analyzed in terms of overall survival (OS), relapse-free survival (RFS), disease-specific survival, and progression-free survival from PAAD. Kaplan-Meier analysis and the log-rank test were used to analyze the results, and *p* < 0.05 was considered statistically significant.

### Statistical analysis

Survival analysis statistics were obtained using the log-rank test, and associations of the NT5DC family with immune infiltration and immune cell type markers were evaluated using Spearman's correlation. The Student's t-test was used to compare two independent samples. Statistical significance was set at *p* < 0.05.

## Results

### Aberrant Expression of the NT5DC Family Members in PAAD

**Figure 1:**
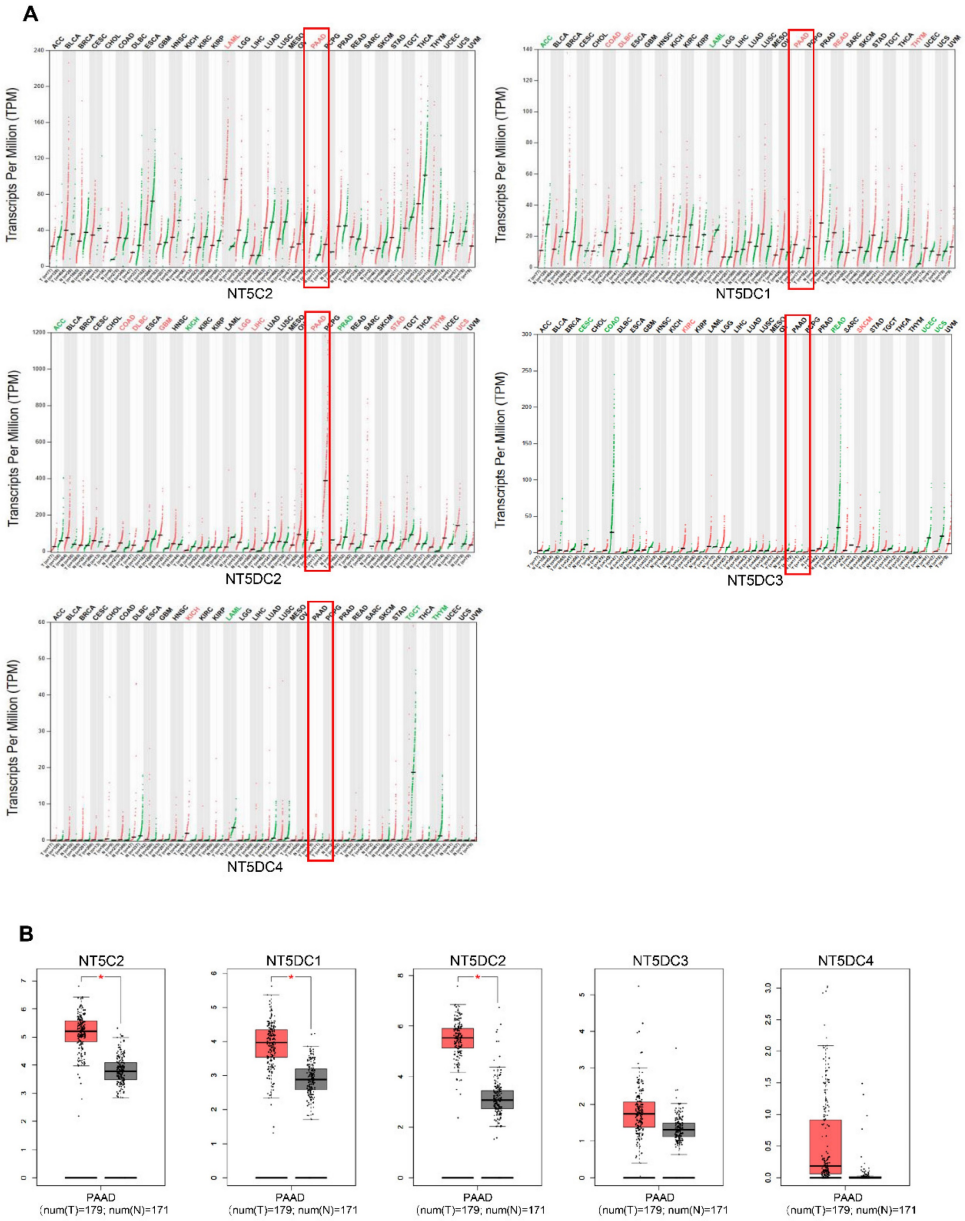
** NT5DC family member expressions in PAAD. (A)** The pan-cancer expression of the mRNAs of NT5DCs is shown. **(B)** The expressions of the NT5DC family mRNA in PAAD are shown. *p < 0.01 compared with control. PAAD, pancreatic adenocarcinoma.

The mRNA levels of NT5DC family members were analyzed using the GEPIA database to compare the expression of NT5DC in different tumors with that in normal tissues (**Figure 1A**). The mRNA expression levels of NT5C2, NT5DC1, and NT5DC2 were upregulated in PAAD tissues compared with those in normal pancreatic tissues (**Figure 1B**).

### Correlation of NT5DC Family Expressions with Clinicopathologic Features of PAAD

**Figure 2:**
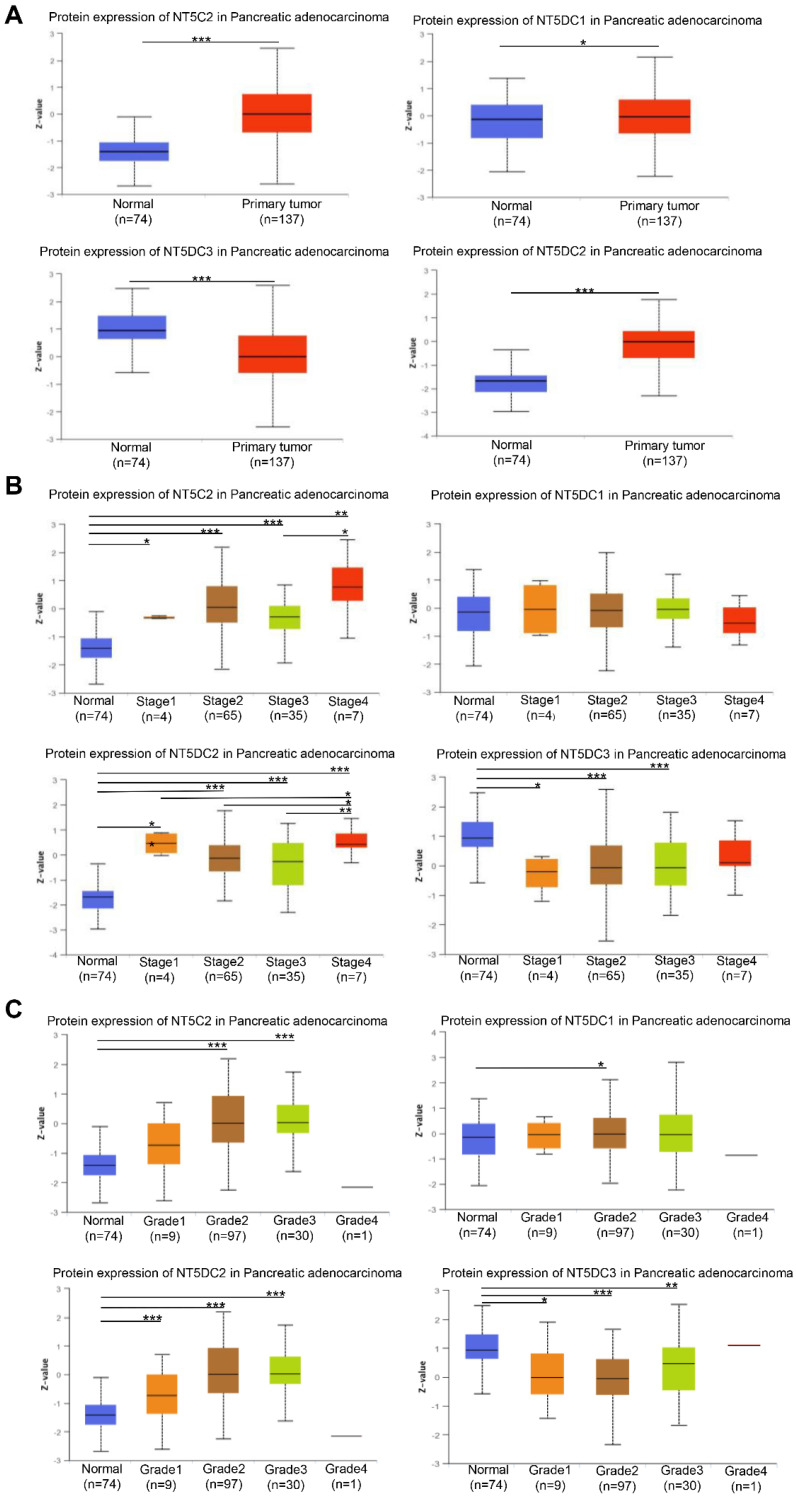
**Relationships between stage and grade of PAAD and NT5DC family member expressions. (A)** The protein expression levels of NT5DC family members in patients with PAAD are shown. **(B)** The correlations between NT5CD family member expressions and stages of PAAD are shown. **(C)** The correlations between NT5CD family member expressions and grades of PAAD are shown. *p < 0.05, **p < 0.01, ***p < 0.001 compared with control. UALCAN, University of Alabama at Birmingham Cancer Data Analysis Portal; PAAD, pancreatic adenocarcinoma.

The expression levels of NT5DC family members were examined to determine whether they correlated with the stage of PAAD. The expressions of NT5C2, NT5DC1, and NT5DC2 proteins were higher in PAAD samples than in normal samples, though the expression of NT5DC3 protein was lower in PAAD samples (**Figure 2A**). The expression levels of NT5DC2/3 proteins were significantly correlated with the cancer stages of PAAD (**Figure 2B**). The expressions of NT5C2 and NT5DC1/2/3 proteins were closely correlated with the grades of cancer (**Figure 2C**).

The relationships between the expression levels of NT5DC family members and the clinicopathological features of PAAD were investigated. The clinicopathological features of 160 patients were downloaded from TCGA database. The mRNA expressions were ranked from lowest to highest and categorized into high and low expression groups based on the median value. NT5DC1 expression correlated with patient sex, NT5DC3 expression was significantly associated with cancer grade, and NT5DC4 expression was associated with tumor diameter (**Table 1**).

### Genetic changes and functional enrichment analysis of NT5DC family members

**Figure 3:**
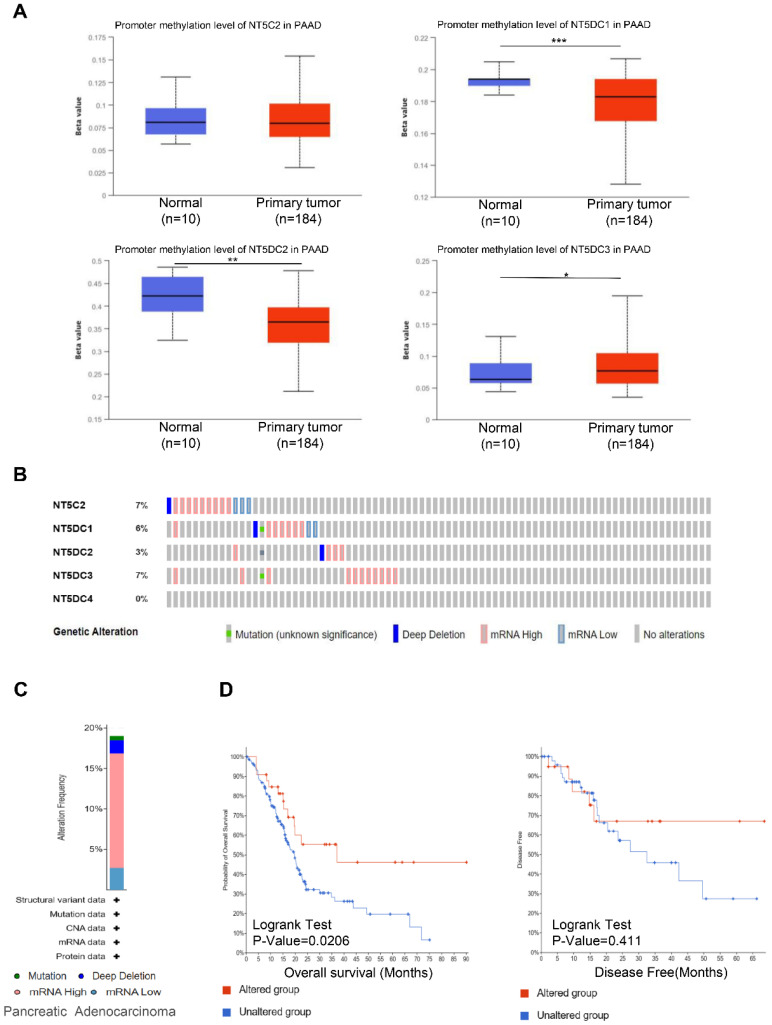
** Genetic alterations and DNA methylation levels of the NT5DC family members in PAAD tissue. (A)** DNA methylation changes in the NT5DC family members in PAAD tissue are shown, as assessed using the UALCAN database.** (B-C)** Summaries of the rate of alteration of the NT5DC family members in PAAD tissue are shown. These were determined using cBioPortal.** (D)** The associations between NT5DC family member mutations and the prognosis of patients with PAAD are shown. *p<0.05, **p<0.01, ***p<0.001 compared with control. PAAD, pancreatic adenocarcinoma; OS, overall survival; DFS, Disease-free survival; UALCAN, University of Alabama at Birmingham Cancer Data Analysis Portal.

Using the UALCAN database, the methylation levels of NT5DC in patients with PAAD were identified. DNA methylation levels of NT5DC1/2 were lower in PAAD samples than in healthy individuals, while NT5DC3 had significantly higher levels in PAAD tissue (**Figure 3A**). The DNA methylation of NT5C2 was not significantly different between normal and PAAD tissues. Genetic variations in the NT5DC family were also investigated. The proportion of genetic variation in NT5DC family members increased from 0% to 7% in patients with PAAD. Mutations in NT5C2, NT5DC1, NT5DC2, NT5DC3, and NT5DC4 were found in 7%, 6%, 3%, 7%, and 0% of the samples, respectively (**Figure 3B**). The most common abnormalities of the NT5DC family in patients with PAAD were mRNA alterations (**Figure 3C**). The associations between NT5DC family member mutations and patient prognosis were also evaluated. Mutations in the NT5DC family members were associated with better OS (**Figure 3D**). However, NT5DC family gene mutations were not significantly correlated with disease-free survival (DFS). These results indicate that mutations in NT5DC family members may serve as potential therapeutic targets, which may significantly improve the prognosis of patients with PAAD.

**Figure 4:**
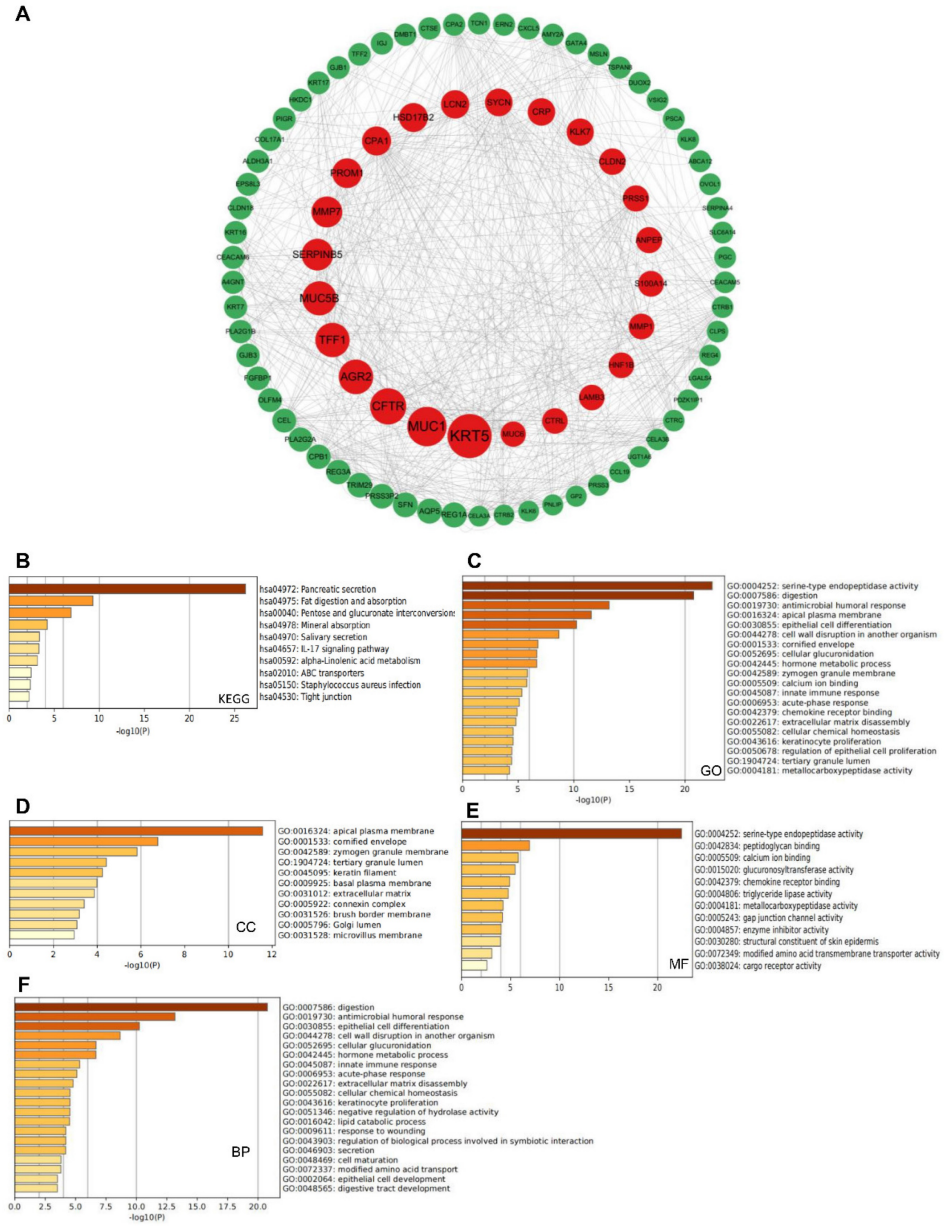
** Predicted functions and signaling pathways of NT5DCs and NT5DC-associated molecules co-expressed in patients with PAAD. (A)** A total of 208 NT5DC-associated co-expressed molecules that are frequently altered in patients with PAAD are identified. The cBioPortal database was used to identify these molecules. The protein-protein interaction network of the NT5DC family members and their associated co-expressed genes, which was constructed using the Cytoscape database, is shown. **(B-F)** A functional enrichment analysis used to analyze the biological functions of NT5DC members and their co-expressed genes is shown. PAAD, pancreatic adenocarcinoma.

A total of 208 co-expressed genes were identified from the cBioportal database and a map of co-expression networks of key genes associated with the NT5DC family members was created using Cytoscape_v.3.7.2 (**Figure 4A**). GO annotation and KEGG pathway analyses were used to analyze the biological functions of NT5DC members and their co-expressed genes using the Metascape database. Pancreatic secretion, fat digestion and absorption, pentose and glucuronate interconversion, and the IL-17 signaling pathway were explored for co-expressed genes (**Figure 4B**). The co-expressed genes were mainly correlated with serine-type endopeptidase activity, digestion, antimicrobial humoral responses, and innate immune responses (**Figure 4C**). Bioprocess analysis indicated that these genes were mainly involved in metabolism, humoral antimicrobial response, epithelial cell differentiation, and innate immune response (**Figure 4D**). Molecular function analysis revealed that the genes were mainly involved in serine-type endopeptidase activity, peptidoglycan binding, and chemokine receptor binding **(Figure 4E)**. Analysis of the cellular components revealed that these genes were frequently associated with the apical plasma membrane and cornified envelope (**Figure 4F**).

### Association Between the Expressions of NT5DC Family Members and Immune Infiltration in patients with PAAD

**Figure 5:**
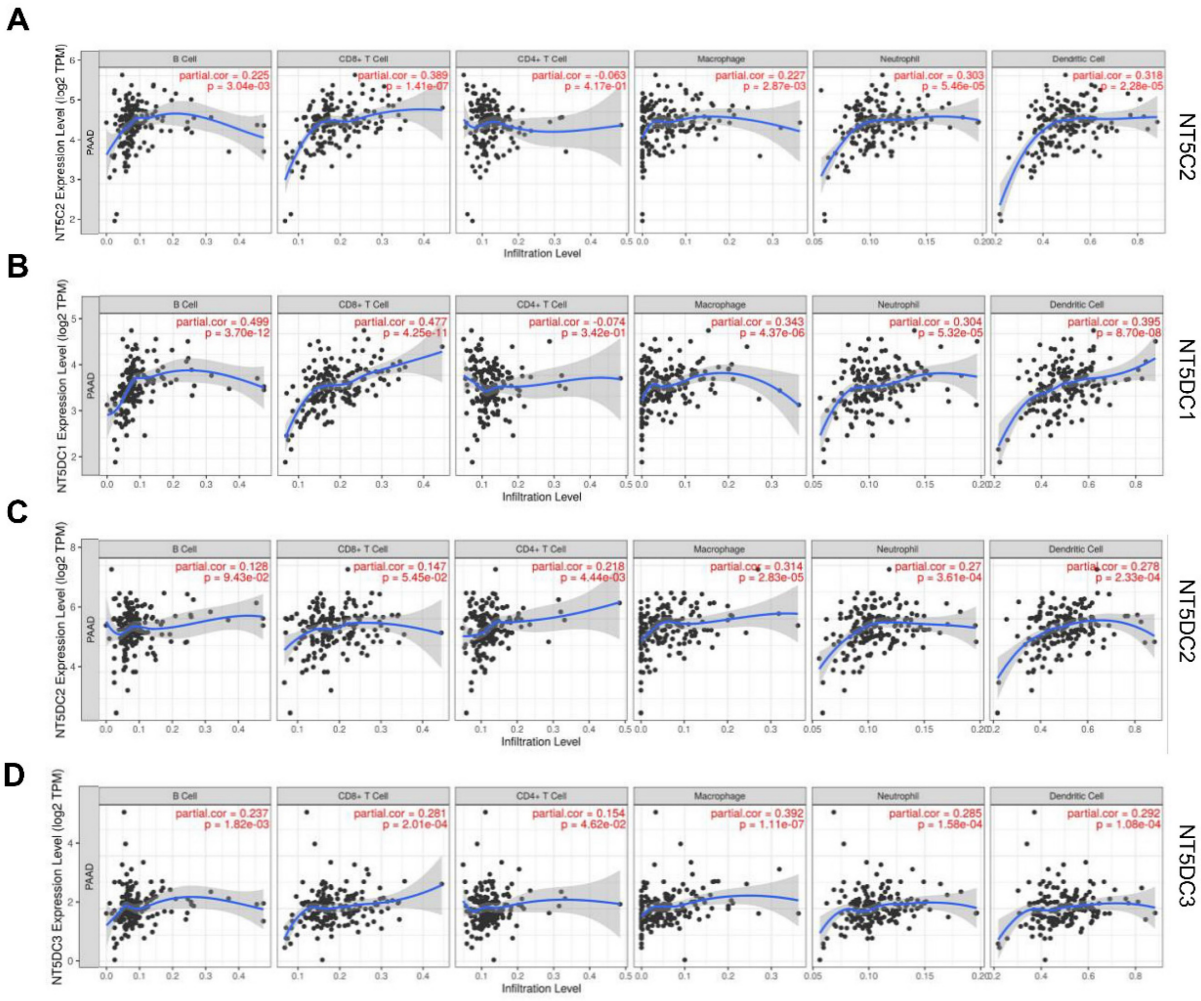
** Association of the expression levels of NT5DC mRNA with the infiltration of immune cells. (A-D)** The associations of NT5C2 and NT5DC 1-3 with immune cell infiltration, determined using the TIMER2.0 database, are shown. TIMER, Tumor Immune Estimation Resource.

TIMER2.0 was used to investigate the correlation between the NT5DC gene family members and immune cell infiltration (**Figure 5**). The expression of NT5C2 mRNA was significantly associated with the infiltration of B cells (correlation coefficient (cor) = 0.225, *p <* 0.05), CD8+T cells (cor = 0.389, *p <* 0.05), and macrophages (cor = 0.227, *p <* 0.05). NT5DC1 expression was significantly associated with B cell (cor = 0.499, *p <* 0.05), CD8+T cell (cor = 0.477, *p <* 0.05), and macrophage (cor = 0.343,* p <* 0.05) infiltration. NT5DC2 expression was significantly associated with CD4+ T cell (cor = 0.218, *p <* 0.05) and macrophage (cor = 0.314, *p <* 0.05) infiltration. NT5DC3 expression was significantly associated with the infiltration of B cells (cor = 0.237, *p <* 0.05), CD8+ T cells (cor = 0.281, *p <* 0.05), CD4+ T cells (cor = 0.154, *p <* 0.05), and macrophages (cor = 0.392, *p <* 0.05).

To further explore the relationship between NT5DC family member expression and different immune cells, the marker types of dendritic cells, CD8+ T cells, neutrophils, and tumor-associated macrophages were analyzed in PAAD using the TIMER database. NT5C2 levels were strongly associated with Th2 and Tregs. NT5DC1 was strongly associated with M2 macrophages, neutrophils, Th17, Tregs, and monocytes and moderately associated with B cells and T cells. There was a moderate or weak correlation between NT5DC2 and B cells, M1 macrophages, M2 macrophages, and Treg markers. The NT5DC3 level was strongly associated with M2 macrophages and the exhaustion of T cells and monocytes (**Table 2**). The NT5DC gene family was implicated in the regulation of macrophage polarization in patients with PAAD, as a clear relationship was found between some components of the NT5DC gene family and M2 macrophage markers.

### Prognostic Values of NT5DC Gene Family Members in Patients with PAAD

**Figure 6:**
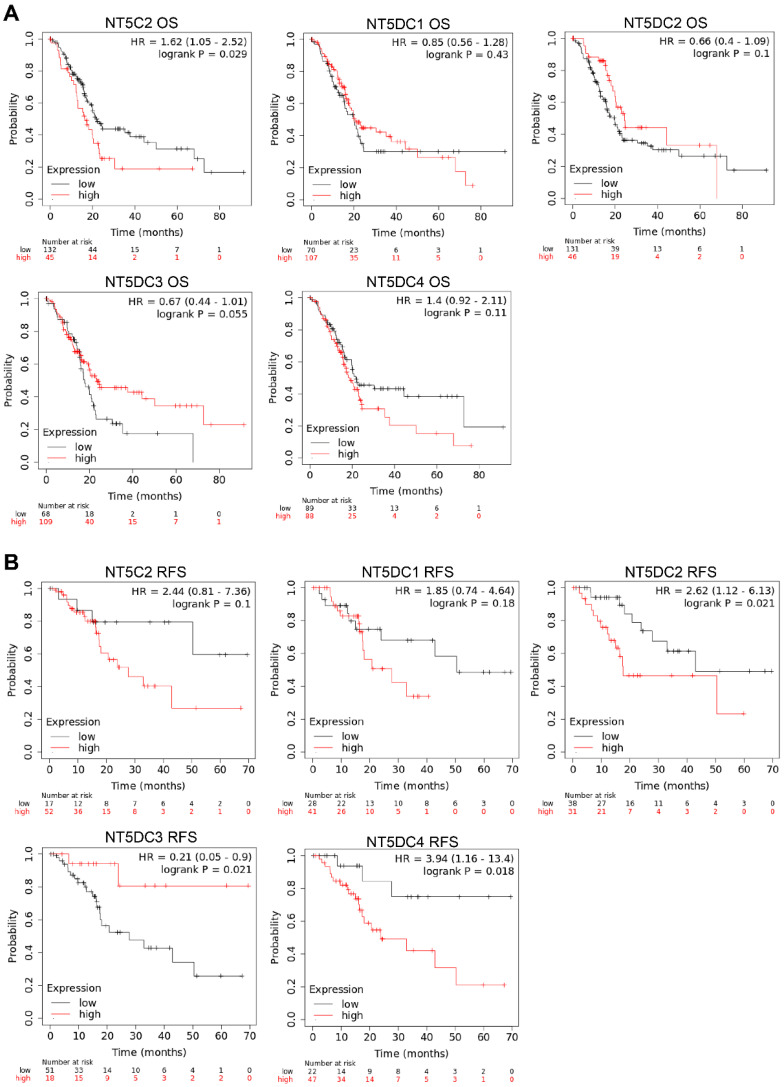
** Prognostic values of mRNA expression levels of NT5DC family members in patients with PAAD. (A-B)** Kaplan-Meier plots showing the OS and RFS of the NT5DC family members in patients with PAAD are shown. PAAD, pancreatic adenocarcinoma; OS, overall survival; RFS, relapse-free survival.

The Kaplan-Meier plotter database was used to investigate the prognostic values of NT5DC family member mRNA expression in patients with PAAD. Higher NT5C2 mRNA expression (OS hazard ratio (HR) = 1.62 (1.05-2.52), *p* = 0.024) was associated with poor OS in patients with PAAD (**Figure 6A**). Increased expressions of NT5DC2 (RFS HR = 2.62 (1.12-6.13), *p* = 0.033) and NT5DC4 (RFS: HR = 3.94 (1.16-13.4), *p* = 0.018) were significantly associated with poorer RFS in patients with PAAD. However, increased expression of NT5DC3 (RFS: HR = 0.21 (0.05-0.9), *p* = 0.021) was associated with better RFS (**Figure 6B**).

**Figure 7:**
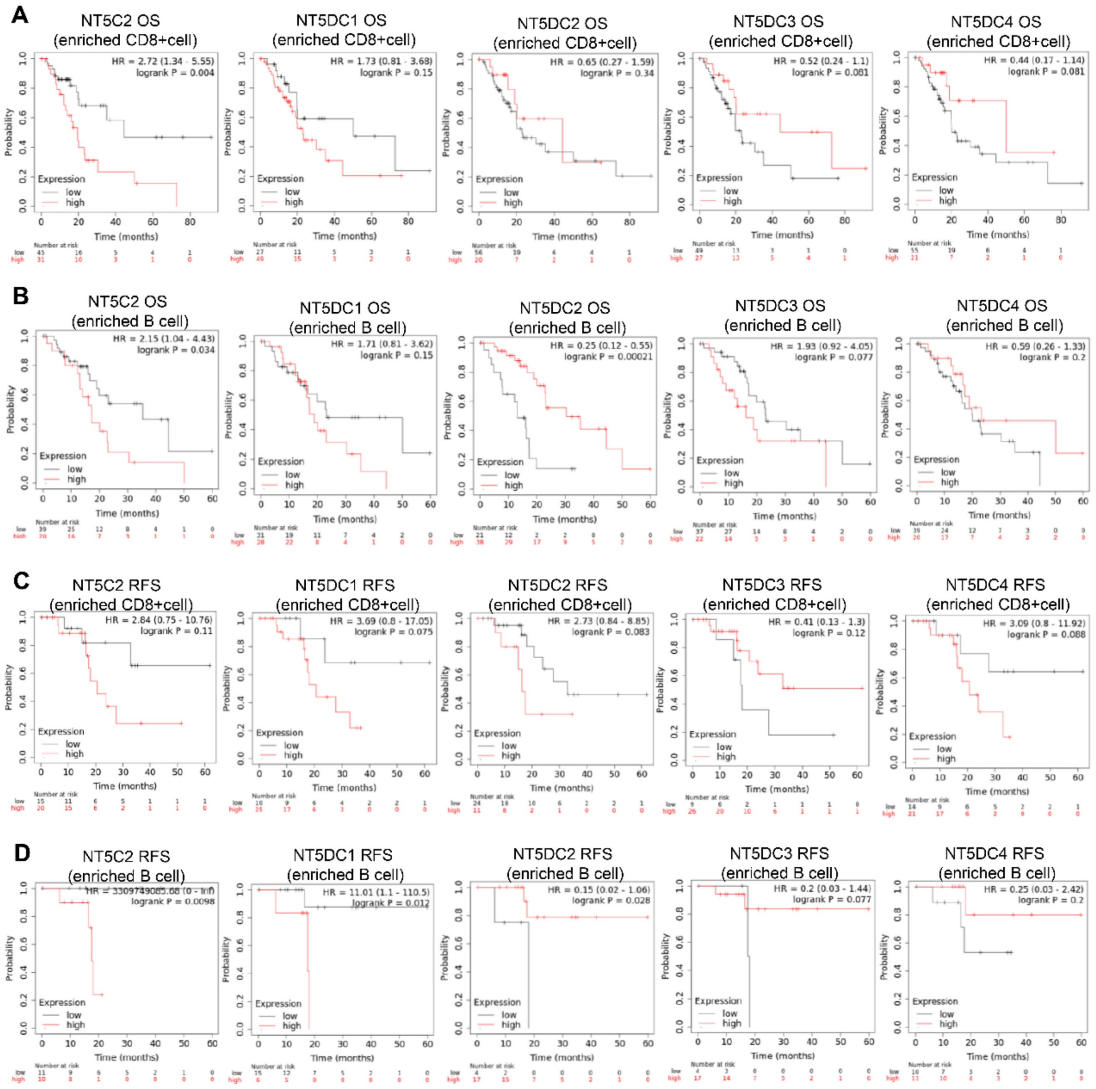
** Prognostic values of the NT5DC family members in relation to immune cell enrichment in patients with PAAD. (A-D)** The prognostic values of the NT5DC family members in relation to CD8+ T cell and B cell enrichment in patients with PAAD are shown. PAAD, pancreatic adenocarcinoma; OS, overall survival; RFS, relapse-free survival.

The prognostic values of the NT5DC family members in relation to immune cell enrichment in patients with PAAD were also investigated. NT5C2 upregulation was associated with poor OS when patients with PAAD were CD8+ T cell-enriched (**Figure 7A**). When patients with PAAD were enriched with B cells, NT5DC1 expression was significantly and negatively correlated with OS and RFS. In contrast, upregulated NT5DC2 expression was associated with favorable OS and RFS in patients with PAAD, and upregulated NT5C2 expression was associated with poor RFS in patients with PAAD with B cell enrichment (**Figure 7B-D**).

## Discussion

PAAD is a major cause of tumor-related death, with high mortality and metastasis rates. Despite improvements in the detection and treatment of PAAD, the prognosis remains poor, with a five-year survival rate < 10% 1, 30. There is an urgent need to identify novel and effective diagnostic and prognostic biomarkers to significantly improve the prognosis of patients with PAAD 31, 32. Intracellular nucleotide hydrolysis is performed by the evolutionarily-conserved 5'-nucleotide enzyme family NT5DC 8. Several studies have reported that members of the NT5DC family are aberrantly expressed in various malignancies and are essential for cancer proliferation and development 13, 33, 34. Unfortunately, the functions of the NT5DC family members in PAAD have not been thoroughly investigated. The biological effects of each NT5DC family member in relation to PAAD were elucidated from five different perspectives in this study: mRNA and protein expression levels, clinical features and disease prognosis, gene mutation, pathway analysis, and immune infiltration. The mRNA and protein levels of NT5C2, NT5DC1, and NT5DC2 were overexpressed in PAAD cells when compared to the expression in non-tumoral cells, whereas the protein levels of NT5DC3 were downregulated in PAAD cells. NT5DC family members can be used for clinicopathological cancer diagnosis, as reported in recent studies. Arik et al. 21 reported that the high expression of NT5C2 could be used as a prognostic marker in patients with lung cancer with poor prognoses. Guo et al. 12 discovered that NT5DC2 promotes the carcinogenesis of glioma stem cell-like cells by upregulating Fyn, and can be used as a potential therapeutic target for glioblastoma.

Therefore, the prognostic significance of aberrantly expressed members of the NT5DC family in patients with PAAD and the clinicopathological associations of these genes were further investigated in this study. The protein expression of NT5DC family members was significantly correlated with the stage and grade of PAAD. In patients with PAAD, overexpression of NT5C2 mRNA was associated with poor OS; overexpression of NT5DC2/4 mRNA was significantly associated with poor RFS; and overexpression of NT5DC3 mRNA was correlated with better RFS. These results suggest that NT5C2 and NT5DC2 may have important prognostic values and great potential as diagnostic markers in patients with PAAD.

Genetic mutations that alter the biochemistry of tissue cells can lead to the development of cancer. Previous studies have reported that variants of the NT5DC family members are involved in tumor development. NT5C2 is associated with chemoresistance and a poor prognosis in patients with relapsed acute lymphoblastic leukemia 35. NT5DC1 polymorphisms are associated with susceptibility to chronic obstructive pulmonary disease 11. The NT5DC gene alteration group (which mainly includes mutations and mRNA changes) is associated with a better OS than the unaltered group. These results are in accordance with the results of the current study regarding NT5DC family gene alterations in PAAD. NT5DC1/2 DNA methylation levels in tumor tissue were lower than those in normal tissue, while the DNA methylation levels of NT5DC3 were significantly increased in tumor tissue, suggesting that DNA methylation-targeting drugs may have applications in cancer treatment 36 as they implicate epigenetic regulation in the biological process of PAAD induced by the NT5DC family. The co-expressed genes of the NT5DC family, including UGT2B7, MT1G, ACADL, MT1H, and others, were found to be associated with pancreatic cancer in previous studies 37-40. Enrichment analyses of co-expressed genes revealed strong correlations between members of the NT5DC family and the antimicrobial humoral response, innate immune response, IL-17 signaling pathway, serine-type endopeptidase activity, and other immune-related signaling pathways. Therefore, the NT5DC family members interact with key molecules in tumor immune-related pathways and should be considered attractive therapeutic targets for the treatment of PAAD.

Pancreatic cancer is a tumor of the digestive tract with a very poor prognosis. The clinical characteristics of pancreatic cancer, including the difficulty of early detection, low surgical resection rate, and high recurrence and metastasis rates after surgery, make it particularly difficult to diagnose 1. Surgical resection is currently the primary treatment for pancreatic cancer; however, patient survival rates are low 41, 42. Pancreatic cancer mortality is largely due to an immunosuppressive environment, inadequate T cell infiltration, and a low mutation burden. However, immunotherapy appears to have a synergistic effect with other treatment modalities and increases response rates. Therefore, the development of multimodal treatments that target the mechanism of resistance to immunotherapy is necessary 43, 44. Li et al. reported that NT5DC2 upregulated Epidermal growth factor receptor (EGFR) expression by downregulating EGFR ubiquitination and preventing ubiquitin-proteasome degradation but not transcription. Upregulating EGFR activated downstream signaling, playing a critical role in the protumourigenic effects of NT5DC2. Furthermore, NT5DC2 expression was associated with larger tumor size and microvascular invasion, and was independently associated with RFS. These findings suggest that NT5DC2 may be a potential molecular target for the treatment of hepatocellular carcinoma 34. Hu et al. 19 reported that TEAD4 binds to the NT5DC2 promoter to activate its transcription. The overexpression of TEAD4 was associated with poor survival, suggesting that NT5DC2 plays an important role in the development of leiomyosarcomas and may serve as a potential therapeutic target. In addition, NT5C2 is involved in metabolizing cladribine, an immunomodulatory drug used to treat multiple sclerosis 45. Low-intensity maintenance therapy with 6-mercaptopurine (6-MP) limits the incidence of acute lymphoblastic leukemia (ALL) relapse, though activating mutations in the NT5C2 gene confer resistance to 6-MP in patients with early relapse of ALL and directly contribute to ALL relapse 46. NT5C2 has been identified as an important therapeutic target in hematological cancers. Zsuzsanna et al. used a combined approach based on fragment-based drug design and *in silico* methods to design potential inhibitors of NT5C2 that act synergistically with known antitumor agents 47. The results of these studies indicate that the NT5DC family has great potential in immunotherapies. This study aimed to identify potential targets for PAAD treatment by investigating the associations between NT5DC family members and immune cell infiltration. The results of this study indicate that immune cells are correlated with the NT5DC family to different degrees. NT5C2 and NT5DC1/2/3 were associated with neutrophils, dendritic cells, and macrophages, suggesting that the NT5DC family may be a good indicator of the immunological health of patients with PAAD. Furthermore, the infiltration of antitumor immune cells, such as CD8+ T and B cells, improves the prognosis of patients with PAAD. The results of the current study demonstrate that NT5C2 and NT5DC2 may be useful immunotherapeutic targets for the treatment of PAAD. However, the current study is limited by the fact that the information used to analyze the data was collected from internet databases. To verify the function and potential molecular mechanisms of NT5C2 and NT5DC2 in PAAD, *in vitro* and* in vivo* experiments and clinical studies are necessary.

## Conclusion

Bioinformatics tools were used to comprehensively evaluate the expression and prognosis of NT5DC family members in patients with PAAD to understand the biological and immunological effects of the NT5DC family in PAAD. The findings suggest that NT5C2 and NT5DC2 may be targets and valuable biomarkers to establish personalized treatments for patients with PAAD, which may help with the development of improved diagnostic and therapeutic modalities to improve patient prognoses. Further research is needed to identify new pharmacological therapies and to evaluate the mechanisms of their effects on carcinogenesis and development.

## Supplementary Material

Supplementary table.

## Figures and Tables

**Table 1:** Clinicopathologic parameters and the expressions of NT5DC family members in PAAD.

Characteristics	*N*	NT5C2			NT5DC1			NT5DC2			NT5DC3			NT5DC4		
		Low	High	*p*	Low	High	*p*	Low	High	*p*	Low	High	*p*	Low	High	*p*
**Gender**				0.405			0.761			0.089			0.127			0.114
Male	89	51	38		48	41		57	32		55	34		56	33	
Female	71	36	35		40	31		36	35		52	19		53	18	
**Age**				0.177			**0.032**			0.522			0.065			0.932
≤55	32	14	18		23	9		17	15		17	15		22	10	
>55	128	73	55		65	63		76	52		90	38		87	41	
**T Stage**				0.246			0.558			0.76			0.446			0.39
T1 + T2	28	18	10		14	14		17	11		17	11		21	7	
T3 + T4	132	69	63		74	58		76	56		90	42		88	44	
**N Stage**				0.641			0.519			0.911			0.102			0.452
NX + N0	47	22	25		24	23		27	20		27	20		30	17	
N1	113	65	48		64	49		66	47		80	33		79	34	
**M Stage**				0.215			0.215			0.164			0.956			0.963
Mx	82	47	35		49	33		52	30		55	27		56	26	
M0 + M1	78	40	38		39	39		41	37		52	26		53	25	
**Stage**				0.743			0.825			0.636			0.16			0.58
StageI	19	11	8		10	9		12	7		10	9		14	5	
StageII+III+IV	141	76	65		78	63		81	60		97	44		95	46	
**Grade**				0.757			0.61			0.336			**0.035**			0.543
Gx + G1	26	15	11		12	14		18	8		19	7		20	6	
G2	86	48	38		49	37		46	40		50	36		58	28	
G3 + G4	48	24	24		27	21		29	19		38	10		31	17	
**Diameter(cm)**				0.951			0.774			0.784			0.383			**0.028**
≤4	107	58	49		58	49		63	44		74	33		79	28	
>4	53	29	24		30	23		30	23		33	20		30	23	

Bold font indicates significant difference.

**Table 2:** Correlations between the expressions of NT5DC family members and the markers of immune cells.

		NT5C2		NT5DC1		NT5DC2		NT5DC3	
		**Cor**	***p***	**Cor**	***p***	**Cor**	***p***	**Cor**	***p***
CD8 + Tcell	CD8A	0.05	0.518	0.251	**0.012**	0.047	0.545	0.217	**0.004**
	CD8B	0.058	0.450	0.185	**0.011**	0.131	0.087	0.08	0.297
	GZMA	0.04	0.602	0.179	**0.019**	0.117	0.128	0.176	**0.022**
B cell	CD19	0.007	0.925	0.155	**0.043**	0.256	**0.001**	0.108	0.160
	CD79A	-0.007	0.932	0.18	**0.018**	0.239	**0.002**	0.082	0.288
	MS4A1	0.013	0.871	0.181	**0.018**	0.174	**0.023**	0.084	0.277
T cell	CD3D	0.038	0.623	0.197	**0.010**	0.14	0.069	0.19	**0.013**
	CD3E	0.064	0.409	0.236	**0.002**	0.12	0.119	0.208	**0.006**
	CD2	0.064	0.407	0.259	**0.001**	0.104	0.176	0.175	**0.022**
TAM	CCL2	-0.004	0.963	0.026	0.735	0.13	0.089	0.134	0.081
	CD68	0.313	**0.000**	0.361	**0.000**	0.174	0.023	0.209	**0.006**
	IL10	0.097	0.209	0.125	0.103	0.21	**0.006**	0.254	**0.001**
M1	IRF5	0.298	**0.000**	0.201	**0.008**	0.197	**0.010**	0.321	**0.000**
	PTGS2	0.297	**0.000**	0.142	0.064	0.315	**0.000**	0.19	**0.013**
	NOS2	0.149	0.051	0.14	0.068	0.226	**0.003**	0.131	0.087
M2	MS4A4A	0.086	0.262	0.284	**0.000**	0.191	**0.012**	0.312	**0.000**
	CD163	0.117	0.128	0.316	**0.000**	0.193	**0.011**	0.365	**0.000**
	VSIG4	0.104	0.176	0.255	**0.001**	0.21	**0.006**	0.308	**0.000**
Neutrophils	ITGAM	0.184	**0.016**	0.265	**0.000**	0.227	**0.003**	0.225	**0.003**
	CCR7	0.068	0.376	0.244	**0.002**	0.141	0.066	0.18	**0.019**
	SIGLEC5	0.143	0.061	0.288	**0.000**	0.236	**0.002**	0.228	**0.003**
DC	HLA-DQB1	0.07	0.364	0.241	**0.002**	0.07	0.360	0.229	**0.003**
	HLA-DPB1	0.035	0.646	0.219	**0.004**	0.205	**0.007**	0.197	**0.010**
	HLA-DRA	0.152	**0.047**	0.318	**0.000**	0.188	**0.014**	0.22	**0.004**
	HLA-DPA1	0.117	0.128	0.327	**0.000**	0.184	**0.016**	0.236	**0.002**
	ITGAX	0.068	0.375	0.134	0.081	0.263	**0.001**	0.247	**0.001**
	CD1C	0.142	0.065	0.285	**0.000**	0.145	0.059	0.13	0.091
	NRP1	0.294	**0.000**	0.3	**0.000**	0.249	**0.001**	0.29	**0.000**
NK cell	KIR2DL1	0.035	0.651	0.119	0.120	-0.038	0.620	0.099	0.199
	KIR2DL3	0.044	0.565	0.018	0.816	0.064	0.407	0.088	0.252
	KIR2DL4	0.163	**0.033**	0.103	0.178	-0.054	0.480	0.148	0.054
	KIR3DL1	-0.048	0.535	0.043	0.575	-0.018	0.814	0.117	0.129
	KIR3DL2	0.0324	0.674	0.164	**0.032**	0.098	0.204	0.137	0.073
	KIR3DL3	0.123	0.108	0.066	0.393	0.067	0.381	-0.02	0.792
	KIR2DS4	-0.068	0.378	0.132	0.086	-0.076	0.325	0.055	0.478
Th1	TBX21	-0.002	0.974	0.174	**0.023**	0.002	0.974	0.212	**0.005**
	STAT1	0.433	**0.000**	0.363	**0.000**	0.06	0.436	0.208	**0.006**
	STAT4	-0.055	0.474	0.165	**0.031**	0.074	0.336	0.271	**0.000**
	IFNG	0.043	0.574	0.099	0.199	-0.025	0.743	0.149	0.052
Th2	STAT6	0.561	**0.000**	0.39	**0.000**	0.023	0.767	0.246	**0.001**
	GATA3	0.215	**0.004**	0.107	0.163	0.263	**0.001**	0.079	0.302
	STAT5A	0.266	**0.000**	0.233	**0.002**	0.219	**0.004**	0.337	**0.000**
Tfh	BCL6	0.392	**0.000**	0.282	**0.000**	0.185	**0.015**	0.356	**0.000**
	IL21	0.035	0.654	0.077	0.316	0.051	0.504	0.079	0.305
Th17	STAT3	0.396	**0.000**	0.43	**0.000**	0.189	**0.014**	0.449	**0.000**
	IL17A	0.031	0.688	0.213	**0.005**	-0.123	0.109	0.071	0.353
Treg	FOXP3	0.164	**0.032**	0.238	**0.002**	0.257	**0.001**	0.194	**0.011**
	STAT5B	0.317	**0.000**	0.403	**0.000**	0.212	**0.014**	0.462	**0.000**
	CCR8	0.284	**0.000**	0.363	**0.000**	0.187	**0.015**	0.231	**0.002**
T exhaustion-cell	PDCD1	-0.053	0.492	0.119	0.122	0.141	0.065	0.217	**0.004**
	CTLA4	0.082	0.286	0.197	**0.010**	0.188	**0.014**	0.285	**0.000**
	HAVCR2	0.13	0.089	0.247	**0.001**	0.218	**0.004**	0.272	**0.000**
	LAG3	-0.061	0.429	0.109	0.157	0.018	0.819	0.31	**0.000**
Monocyte	CD86	0.14	0.068	0.256	**0.001**	0.206	**0.007**	0.257	**0.001**
	C3AR1	0.113	0.143	0.281	**0.000**	0.186	**0.015**	0.27	**0.000**
	CSF1R	0.087	0.257	0.285	**0.000**	0.169	**0.028**	0.302	**0.000**

Bold font indicates significant difference.
